# Research progress on composite material of bismuth vanadate catalyzing the decomposition of Quinolone antibiotics

**DOI:** 10.1038/s41598-024-51485-x

**Published:** 2024-01-18

**Authors:** Yuan Zhao, Lingyuan Kong, Shangdong Li, Zhirui Zhao, Na Wang, Yunqing Pang

**Affiliations:** 1 School of Stomatology, Lanzhou University, 199 Donggang West Road, Lanzhou, Gansu, People’s Republic of China; 2 School of Clinical Medicine Gansu University Of Chinese Medicine, 35 Dingxi East Road, Lanzhou, Gansu, People’s Republic of China; 3 School of Clinical Medicine, Lanzhou University, 199 Donggang West Road, Lanzhou, Gansu, People’s Republic of China

**Keywords:** Structural biology, Biochemistry, Environmental sciences, Materials science

## Abstract

Since quinolone is a kind of synthetic broad-spectrum antibacterial drugs, with the widespread use of this class of antibiotics, the risk and harm to human health have been attendant to the sewage containing quinolones which are discharged into the environment. Photocatalysis is considered as a promising technology for antibiotic degradation for its strong redox properties and reaction rate. As a metal oxidizing substance, Bismuth vanadate (BiVO_4_) is such a popular and hot material for the degradation of organic pollutants recently due to its good photocatalytic activity and chemical stability. Numerous studies have confirmed that BiVO_4_ composites can overcome the shortcomings of pure BiVO_4_ and cleave the main structure of quinolone under photocatalytic conditions. This paper mainly outlines the research progress on the preparation of BiVO_4_ composites and the degradation of quinolone antibiotics from the perspective of improving the catalysis and degrading the efficiency mechanism of BiVO_4_ composites.

## Introduction

The discovery and use of antibiotics have been hailed as one of the greatest achievements in the history of science and technology in the twentieth century. Since antibiotics have been widely used in medicine, animal husbandry, agriculture^1^, it was reported in Brown’s study that global consumption of antibiotics has increased by 46% since 2000^2^. Unfortunately, most antibiotics cannot be fully metabolized in the body of humans or animals, and only some of the excreted antibiotics can be broken down in sewage treatment plants, while the rest goes directly into the environment in its maternal form^3,4^. The antibiotics released into the natural environment are extremely hazardous, inducing the formation of drug-resistant bacteria and enriching them in the natural environment, posing a great threat to the balance of the natural ecological chain and the health of humans themselves^5^. In consequence, it is urgent to solve the problem of antibiotic pollution in the water environment and accelerate the development of antibiotic degradation technology.

For its features of high efficiency, energy saving and environmental protection, semiconductor-based photocatalysis technology can use inexhaustible and clean sunlight as an energy source, which has become the focus of many scholars' researches in the past decade^6^. Early on, titanium dioxide (TiO_2_) was used as photocatalyst for the degradation of antibiotics, but the band gap of TiO_2_ is too large, and it can only respond to UV light, which failed to meet the requirements for photocatalytic efficiency^7^_._ In 1998, Kudo et al.^8^ reported for the first time that BiVO_4_ can catalyze hydroxyl radicals (·OH) and superoxide ion radicals (O_2_−) in AgNO_3_ aqueous solution with strong oxidation under visible light irradiation, and reduce organic matter in wastewater into CO_2_, H_2_O, thus achieving organic matter degradation. The smaller the band gap of the material, the higher the catalytic efficiency and the larger the number of electron–hole pairs^6^. However, the relatively large band gap of pure BiVO_4_ led to narrow visible light absorption range, high photo-generated carrier recombination rate, low specific surface area, poor electrical conductivity, and thus it couldn’t meet the requirement of higher redox ability^9^. Subsequently, researchers began to focus on BiVO_4_ composite preparation, using noble metal deposition, doping, hetero-junction, Z-scheme, etc. to construct new structures and improve the catalytic degradation efficiency. Nevertheless, there is a lack of overall information about the practical use of BiVO_4_ composites in photocatalytic degradation. Taking quinolone antibiotics as an example, this paper will firstly review the basic properties of BiVO_4_ composites and then discuss the mechanism of photocatalytic degradation of BiVO_4_; finally, it will focus on the principles of composite degradation of quinolone antibiotics, and discuss the difficulties faced in the photocatalytic degradation process as well as the research and development prospects in this field.

## Basic properties of BiVO_4_ and BiVO_4_ composites

### BiVO_4_

As a low-carbon and environmentally friendly inorganic chemical, BiVO_4_ is non-toxic, narrow band gap and corrosion resistant^10^. The experimental evidence shows that Bi in monoclinic scheelite system (s-m) is more prone to single-pair distortion under visible light irradiation, and has higher photocatalytic oxygen evolution activity in water, as well as good photocorrosion resistance and chemical stability^11,12^. Therefore, the monoclinic BiVO_4_ is usually used in photocatalytic degradation^12^.

The monoclinic BiVO_4_ with a narrow band gap of 2.4 eV which has the ultraviolet absorption band and the visible light absorption band. The visible light absorption is attributed to the transition from a valence band formed by Bi_6s_ or a hybrid orbital of Bi_6s_ and O_2p_ to a conduction band of V_3d_^10,12^. This confluence of factors engenders superior electron dispersion and augmented electron mobility. These properties make monoclinic BiVO_4_ as one of the preeminent and auspicious photocatalysts in contemporary research and applications^13^.

### BiVO_4_ composites

The BiVO_4_ composite material should have the following characteristics: (1) narrow band gap, overlapping the redox potential of water at the energy level to decompose water into H_2_ and O_2_; (2) high electron mobility, rapid migration to the material surface under photocatalysis, and low compounding rate of electron–hole pairs; (3) strong light absorption ability, large surface area and porous, which can enhance water adsorption; (4) economic, environmental protection, efficient and stable^14^. Aiming at the above requirements, for the preparation of BiVO_4_ composite, the current researchers mainly focus on noble metal deposition, metal/nonmetal doping, hetero-junction, Z-scheme and so on.

#### Deposition of noble metals

As for the presence of a large number of free electrons in noble metals^15^, based on the isoelectronic resonance effect, when light matching the electron vibration frequency is irradiated on the metal surface, electron waves will be generated and thus enhancing the absorption of visible light and improving the catalytic efficiency^16^. In addition, when the noble metals are combined with semiconductors (e.g.BiVO_4_), Schottky barriers can be constructed^17,18^, and electrons at the interface flow to the noble metal, generating a built-in electric field, which accelerates electron migration on the one hand, and effectively hinders electron–hole recombination and improves the separation rate on the other Ji et al.^19^ prepared Pd/AgBr/3D-BiVO_4_ by sol deposition-adsorption method, and electron microscopy showed that Pd formed small nanoclusters on the low crystallinity surface. The composite showed as high as 100% degradation of 4-chlorophenol at 140 min. This can be attributed to the nanostructure that effectively transfers separated electrons, and the porous surface provides sites for the attachment of organics.

Noble metal deposition is an effective way of photocatalytic elimination of pollutants, which can be loaded at laboratory temperatures without forming any second phase; meanwhile, the size of the deposited metal can be adjusted by controlling the reaction conditions during the preparation process^20^. However, noble metals are costly, so they are not used on a large scale.

#### Metal/non-metal doping

Metal doping is the insertion of 3d transition elements (e.g. Cu) into the original band gap of BiVO_4_ to form a new impurity energy band. The presence of impurity energy bands reduces the band gap distance, which helps to increase the interfacial electron transfer rate while expanding the absorption of light energy^21,22^. Non-metal doping is using doped non-metals to replace O^2-^ in the lattice of BiVO_4_, resulting in lattice expansion, particle size reduction and surface area increasing, thereby improving the electron transfer rate, increasing the chance of contact between the contaminant and the catalyst, and facilitating the reaction^23^. Wang et al.^24^ combined gC_3_N_4_, CQs with BiVO_4,_ which showed good degradation potential with a degradation rate of 0.0293 min^−1^ for tetracycline hydrochloride under light irradiation. The doping marginalized the valence band of BiVO_4_ and shortened the transfer distance of electron–hole pairs, thus improving the separation efficiency. As seen by the transient photocurrent response^10^, the photocurrent will rise instantaneously when the light source is turned on, whereas the photocurrent will be zero when the light source is turned off. It is proved that the electron conduction velocity of composite material is so fast.

Although doping is an important means of material modification, it also has certain disadvantages, such as decreasing in the number of carriers, thermal instability, etc^21^. It has been found that the formation of impurity energy band will lead to a large gap in the composite material, and the electron–hole pairs that should be in this position will no longer exist, so the catalytic efficiency will be reduced.

#### Heterogeneous junction

A heterojunction is the coupling of two different semiconductors. There are three main types of heterojunctions, among which type II heterojunctions have one semiconductor with lower valence band and conduction band edges than the other, and electrons and holes are confined in different semiconductors, since the heterojunctions demonstrate good ability of electron–hole pair separation, they are widely used in photocatalysis^23^. Cui et al.^25^ constructed CuS-BiVO_4_ p-n heterojunctions and substantiated that the heterojunction could improve the separation of photogenerated carriers and degrade ciprofloxacin under visible light irradiation. Chen et al.^26^ constructed a heterojunction photocatalyst AgI-BiVO_4_ for tetracycline degradation, and the composite has higher photocatalytic performance than pure BiVO_4_.

The valence band (VB) and conduction band (CB) of material A in the heterojunction are higher than that of material B, which drives electrons from A (CB) → B (CB), and holes from B (VB) → A (VB). The electrons are then excited to reduce H^+^ to H_2_ and holes to oxidize H_2_O to O_2_^23^. Although this transfer mode can improve the charge interface separation, the reduction property is reduced due to the migration of electrons from high reduction potential to low reduction potential; similarly, the oxidation property is also reduced due to the migration of holes from high oxidation potential to low reduction potential. Furthermore, during the reaction, as electrons and holes keep migrating, repulsive forces will arise between the same kind and hinder the migration.

#### Z-scheme

The Z-scheme is an improved method based on heterojunctions, belonging to special type of heterojunctions. The catalytic efficiency of semiconductor photocatalysts is determined by its light absorption capacity, electron–hole pair separation, and electricity charge’s directional migration toward the reaction interface^27^. The principle of Z-scheme is combining material B (CB) with low reduction potential binds directly with material A (VB) with low oxidation potential, eliminating electron mediator and directly transferring of photogenerated electrons between semiconductors^27^ Based on this principle, the direct Z-scheme photocatalytic system prepared by combining two different narrow bandgap semiconductors can overcome the repulsion between electrons or holes and indirectly avoid the reduction of redox performance due to the direct combination of low redox potentials. In addition, the Z-scheme can increase the roughness of the material surface. The relatively large specific surface area and porous structure allow the incident light to be reflected in the material for several times thus increasing the absorbance of the material^28^.

By using the unique three-dimensional nanocubic structure of α-Fe_2_O_3_, Ma et al.^29^ prepared Z-Scheme BiVO_4_ /α-Fe_2_O_3_ which showed excellent catalytic degradation activity for antibiotics due to the narrow band gap of the composite and good light absorption ability. The degradation rate of tetracycline was up to 75.8% at 120 min under light. The study of Z-scheme provides a promising avenue for the future design of photocatalysts with special structures.

## Basic mechanism of BiVO_4_ composite photocatalysis

**Figure 1:**
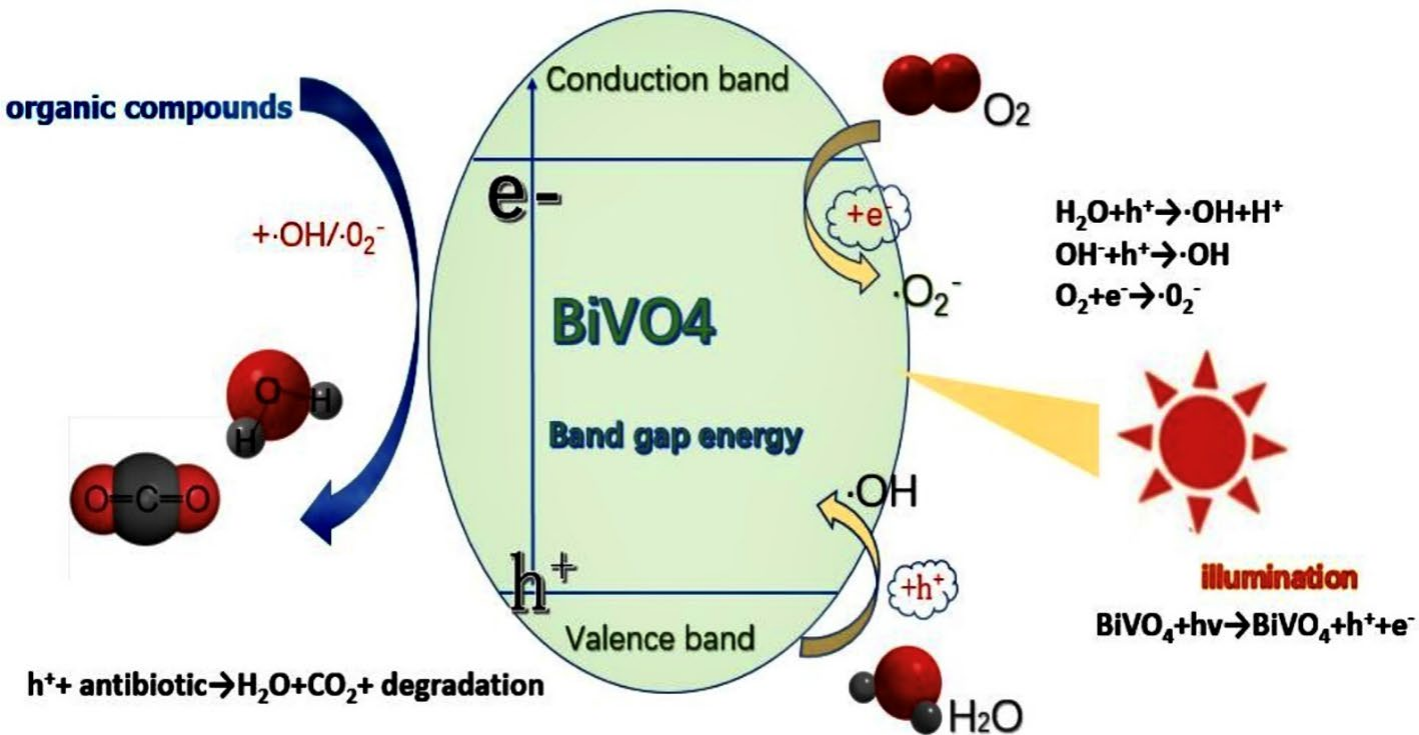
Schematic diagram of the mechanism of photocatalytic degradation of antibiotics.

The main mechanism of BiVO_4_ photocatalyst for antibiotic degradation can be summarized as absorption of photons, excitation, reaction^30^ (Fig. 1).

Specifically, when the photocatalyst receives light irradiation, the electron absorbs energy and transfers from VB to CB, and the corresponding hole is retained in VB^31^. Subsequently, the photogenerated carriers migrate to the surface of the photocatalyst and undergo redox reactions. The hole in VB reacts with H_2_O and OH^−^ to form ·OH. Similarly, electrons in CB react with O_2_ to generate ·O_2_^−^, and the redox system composed of such free radicals has strong redox ability to antibiotics in water^32,33^.

In order to improve the efficiency of organic degradation, researchers used ultrasound-photocatalysis, photoelectrocatalysis and Fenton-like system to improve the catalytic efficiency.

### Ultrasound-photocatalysis and photoelectrocatalysis

Fan et al.^34^ combined conventional ultrasonic catalysis and photocatalysis together, and the combined degradation rate of acousto-optic catalysis could be increased to 84.02%. Due to the combination of these two, the existing defects can be compensated in the single system for catalysis, and then reduce the blockage of the active site of the material, and improve the efficiency of electron transfer. On the other hand, during the acousto-photocatalytic degradation process, the phenomenon of acousto-luminescence has been generated. Under ultrasound radiation, a large number of light waves are excited, which generate reactive oxygen and promote the oxidation reaction^35^.

Photoelectrocatalysis is the most common method to increase the efficiency of catalysis, which is the improvement of photocatalysis. Its principle is that the number of photogenerated charges increases accordingly in the presence of an applied extra electric field; at this point, the photocurrent density inside the composite increases, and the stability of charge separation and transfer is enhanced. The cathode of the composite has enough negative CB to reduce water to H_2_ by electrons; while the anode has enough positive VB to oxidize water to O_2_ by holes^36^. The experimental results showed that the photoelectrocatalytic degradation efficiency of norfloxacin (NOR) by 808 mol M-BiVO_4_/T-BiVO_4_ at 150 min and 2 h has been demonstrated up to be 91%^37^.

### Fenton-like system

The essence of the conventional Fenton method is that the chain reaction between divalent iron ions and hydrogen peroxide catalyzes the formation of ·OH, followed by a redox reaction. However, this reaction can only be carried out in an acidic environment, and is overly dependent on the concentration of H_2_O_2_ and Fe^2+^^38^. In order to ensure the smooth catalytic degradation, the researchers added light to the Fenton system, to use the reduction reaction to reduce the oxygen on the cathode to H_2_O_2_, and to react with Fe^3+^ which was reduced to Fe^2+^ to form ·OH^39,40^. At the same time, ·OH reduces Fe^3+^ to Fe^2+^ under light, which promotes the regeneration of the catalyst^41^. On the other hand, Fe forms more reactive complexes with carboxylic acid residues to transfer electrons to the metal, which decompose to Fe^2+^ and ·OH in the light^42^.

Fan et al.^34^ prepared FeVO_4_-BiVO_4_ to degrade levofloxacin (LFX), during the reaction Fe^3+^, V^5+^ and H_2_O_2_ undergoed a Fenton-like reaction to form hydrogen superoxide (HO_2_)^35,43^. HO_2_ can attack LFX and eventually oxidize it to form low molecular weight organic compounds or mineralize it to inorganic compounds^44^. Lai et al.^44^ tested that the removal rate of ciprofloxacin (CIP) by graphite felt-doped BiVO_4_ (GF-BiVO_4_) was 99.8% under the solar-optic-electric Fenton system. It is speculated that the higher oxygen content and active site of the complex may result in a significant increase in the production of ·OH^45^. In addition, trivalent iron formed a chelate with CIP, and the ring-opening reaction at the chelate site led to an increase in the production of Fe^2+^^46^, with the degradation rate being significantly higher.

## Advances in the application of BiVO_4_ composites in the degradation of quinolone antibiotics

Quinolone antibiotics cause bacteria to fail to reproduce by inhibiting the gyrase enzymes required in the process of bacterial DNA synthesis. There are four generations, and the third generation is more widely used in the clinic, such as NOR、LFX, etc. (see Table 1). In order to better exploit the advantages of BiVO_4_ complex photocatalysis, the degradation mechanism of this material for quinolone antibiotics and the influencing factors need to be studied. In this section, the whole process of photocatalytic degradation of BiVO_4_ composite will be described in details, taking quinolone antibiotics as an example.

**Table 1:** Overview of quinolone antibiotics.

Name	Structure	Antibacterial spectrum	Features
LFX		Gram-negative, Gram-positive-bacteria, Pseudomonas aeruginosa, Mycoplasma, Chlamydia, Mycobacterium tuberculosis^47^	Enhanced inhibition of Gram-positive bacteria by alkyl groups on the piperazine ring^48^
NOR		Infections of the urinary tract, gastrointestinal tract, and respiratory tract caused by Gram-negative bacilli^47^	(1) Improve bioavailability^49^ (2) Inhibition of exclusion mechanism
CIP		Gram-negative, Gram-positive bacteria, Pseudomonas aeruginosa, Mycoplasma, Chlamydia and other atypical bacteria^50^	(1) Piperazine ring improves potency^51^ (2) Cyclopropyl enhances activity^52^
OFL		Most Enterobacteriaceae, Gram-negative bacteria, Gram-positive bacteria and Mycoplasma pneumoniae, Chlamydia pneumoniae^47^	Same as LFX

### Mechanism of degradation of quinolone antibiotics by the BiVO_4_ composites

**Figure 2:**
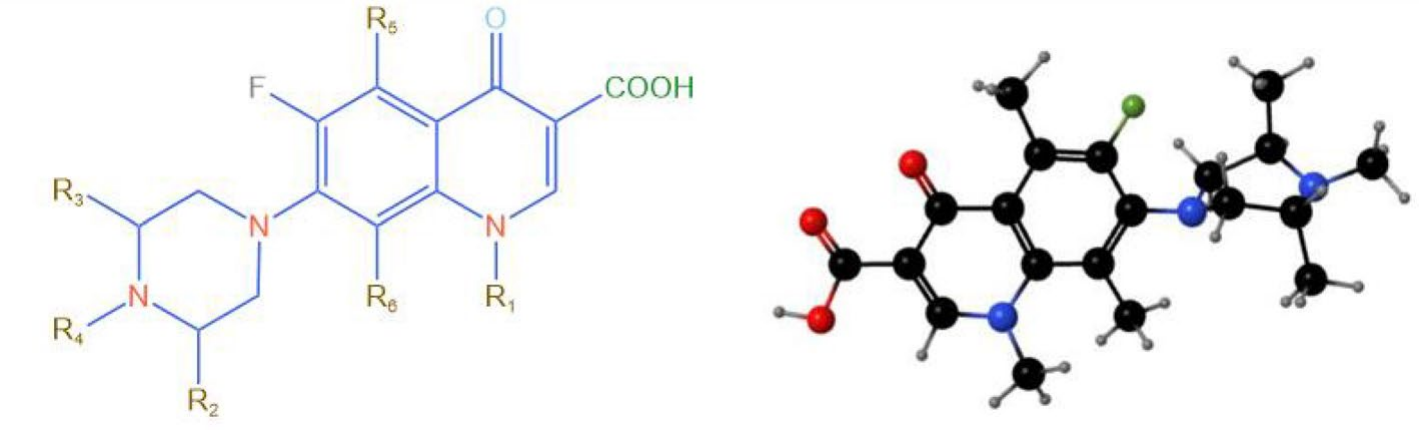
Structural formula of quinolone.

The main chemical structure of quinolone antibiotics is the piperazine ring (as in Fig. 2), which is also the main structure for photocatalytic degradation at present. Ding et al.^53^ found that the high electron density around the piperazine ring is the main adsorption and oxidation site. In addition, due to the electron-rich property, the functional groups of the piperazine ring are distributed with holes, which will react with O_2_ and H_2_O during the hole transfer process, leading to the carbonylation and carboxylation of the piperazine ring^54^. Notably, the addition of metallic materials to BiVO_4_ helps quinolones to expose N–H and COO-bonds, and enhance the adsorption with the photocatalyst^55^. The above characteristics have provided a good basis for the degradation of quinolones by BiVO_4_ composites.

### Degradation of different quinolones by the BiVO_4_ composites

#### LFX

LFX is a common Gram-negative inhibitory antibiotic^56^. Lu et al.^57^ prepared BiVO_4_ -CeVO_4_ based on the similar chemical structure of CeVO_4_ and BiVO_4_. The experimental results showed that the degradation rate of this material to LFX was 95.7% after 5 h’irradiation. Spectroscopic analysis revealed that since CeVO_4_ (CB) is more negative, the e^-^flow from CeVO_4_ (CB) to BiVO_4_ (CB) and the migration of h^+^ from BiVO_4_ (VB) to CeVO_4_ (VB), and the heterojunction formed by the similar structure helped to increase the surface area of the compound, exhibiting more active sites in the catalytic process, and reducing the recombination rate of electron–hole pairs^58^. In addition, the special structural Z-scheme of the heterojunction can facilitate the effective transport of reactants and products. Ma et al.^59^ synthesized Ag_3_ PO_4_ -BiVO_4_ , whose unique rough porous spherical structure not only helped to improve the dispersion of LFX on the catalyst surface, but also increased the active center to facilitate the reaction. The degradation rate of LFX by this technology was 92.44% for 180 min. Fan et al.^34^ used hydrothermal method to synthesize FeVO_4_ /BiVO_4_. BiVO_4_ has energy level in conduction, and its VB and FeVO_4_ form a composite photocatalyst interface which can effectively improve the electron transfer rate^60^. The experimental evidence showed that the degradation rate of this composite material on LFX can reach 97.7%.

**Figure 3:**
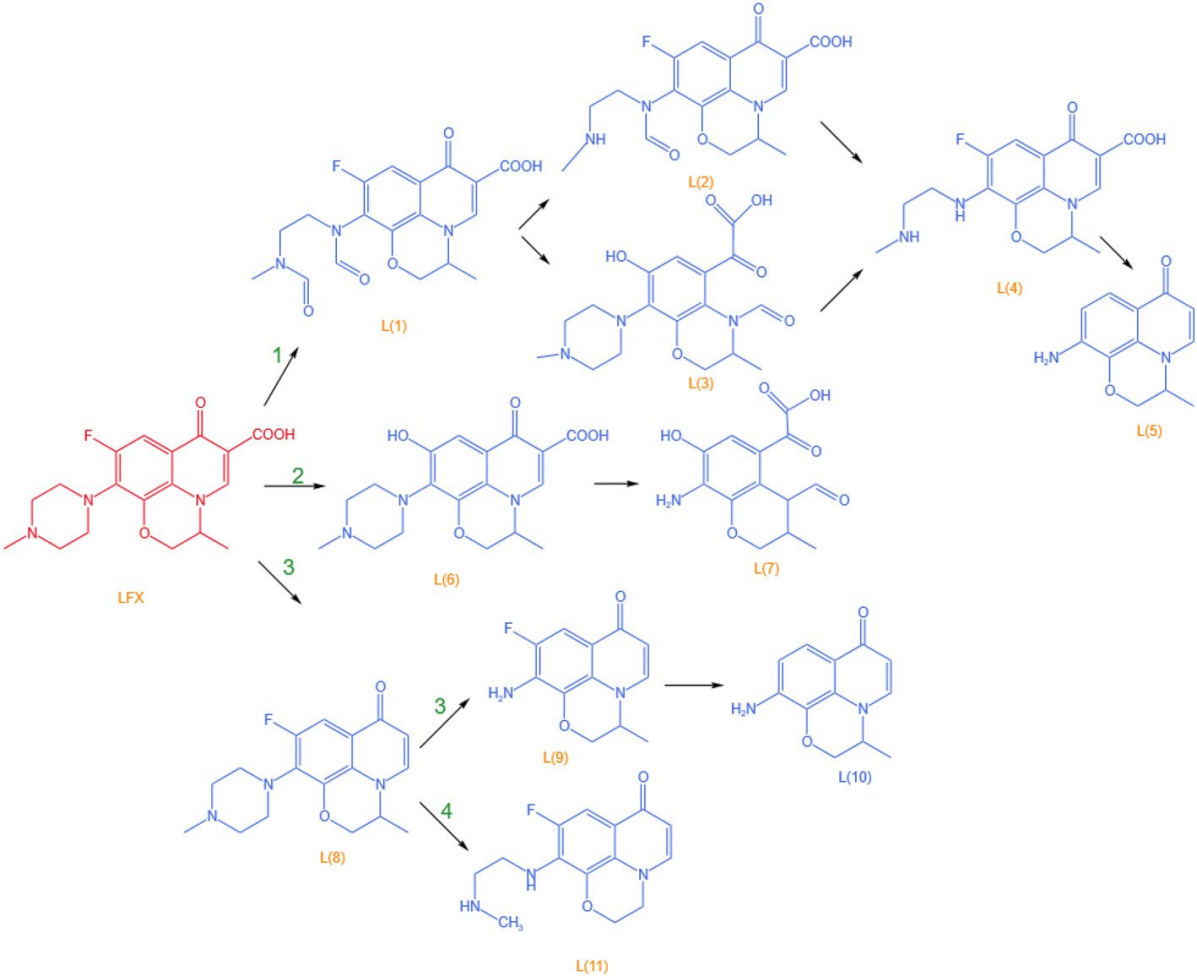
LFX degradation mechanism^34^_._ Copyright 2022 Environmental Research.

The degradation mechanism of LFX is shown in Fig. 3, in which involved the main piperazine ring cleavage, defluorination reaction, decarboxylation reaction and demethylation reaction^34^. Approach 1 firstly is that ·O_2_^-^ attacks the piperazine ring to make it cleaved, and under the attack of ·OH, decarbonylation or hydroxyl substitution generates L2, L3 and L4; finally, the piperazine ring cleaves and the benzene ring F is removed to generate the simple compound L5. Approach 2 is ·OH attacks the benzene ring and substitutes F; subsequently, the benzene ring and piperazine ring are successively destroyed to generate by-products. As for approach 3, the decarboxylation reaction gives rise to L8; thereafter, the piperazine ring decomposes under the oxidation of the active substance, and the defluorination reaction occurs on the benzene ring to generate L10. Approach 4 also involves the cleavage of the piperazine ring after the decarboxylation reaction to generate L11.

#### NOR

As a third generation quinolone antibiotic, NOR can inhibit bacterial DNA synthesis and is commonly used to treat enteritis and dysentery.

To solve the problem of slow separation and fast recombination of electron–hole pairs in monoclinic crystalline BiVO_4_, Baral et al.^37^ designed M-BiVO_4_ /T-BiVO_4_ homoheterojunction, and the degradation rate of this material to NOR reached 91%. Electron microscopy shows that the two semiconductor materials are tightly bonded with well-matched energy band structures, and the electrons are able to migrate directly from the monoclinic phase to the tetragonal phase, which provides the driving force for the spatial separation of photogenerated carriers^61^. Cao et al.^62^ synthesized Ag_3_PO_4_-BiVO_4_ thin film electrode with tin fluoride oxide substrate, and the thin film electrode has high activity of photoelectrochemical decomposition of water. Scanning electron microscopy shows that the composite material has a three-dimensional nanoporous structure and a high photocurrent under light illumination. Under photoelectrocatalysis, the degradation rate was up to 100% for NOR at 90 min. The phosphate-doped BiVO_4_/graphene quantum dots/phosphorus-doped g-C_3_ N_4_ (BVP/GQDsPCN) synthesized by Wang M^63^ et al. also showed good degradation results with 86.3% degradation rate at 120 min. The Z-scheme can promote electron transfer through GQDs as electron mediators and improve the redox potential, thereby block the formation of highly toxic intermediates. This provides an idea for photocatalytic degradation of intermediates.

**Figure 4:**
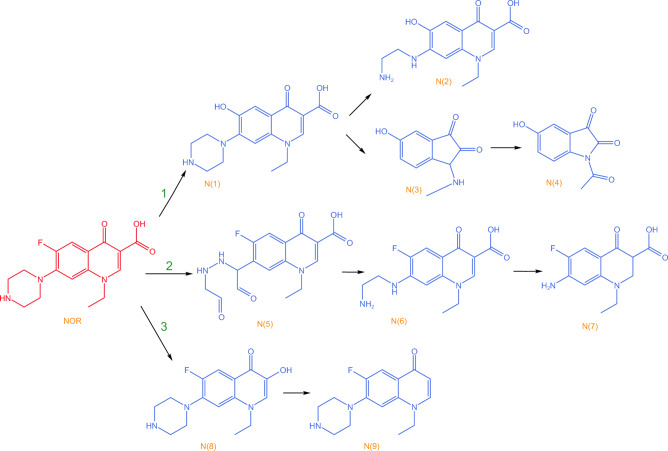
NOR degradation mechanism^63^. Copyright 2021 Separation and Purification Technology.

The specific degradation mechanism of NOR is shown in Fig. 4, whose approaches include: degradation of the piperazine ring, decarboxylation reaction, and hydroxylation reaction^63^. In approach 1, firstly, hydroxylation reaction occurs on the benzene ring; subsequently, N1 piperazine ring is cleaved to generate N2, N3, and N4 is produced under the attack of ·OH. Approach 2 is that the piperazine ring is attacked by ·OH twice successively to generate N5, and N5 undergoes decarboxylation reaction to generate N6, then N6 benzene ring is attacked by ·OH to generate N2, or the piperazine ring is directly broken to generate N7. Regarding approach 3, decarboxylation is converted to N8, followed by dehydroxylation to generate N9.

#### CIP

CIP has superior antibacterial ability and fairly strong exterminating effect to Gram-negative bacteria, especially Pseudomonas aeruginosa.

Due to its high stability and narrow bandgap^64^, gC_3_N_4_ has been widely used in photocatalytic materials for forming Z-scheme by binding with BiVO_4_ as well. Typically, Ma et al.^65^ synthesized pt@ BiVO_4_–gC_3_N_4_ composites, and spectral analysis found that P-conjugate pores were formed between gC_3_N_4_ and BiVO_4_, and the C–N, V–O and Bi–O bonds became longer, which contributed to the separation of charge carriers. Under the condition of 120 min illumination, the degradation rate can reach 100%. rGO is modified on the basis of GO, which has a lower band gap and can enhance the absorption of visible light. The surface of ZnO has a large number of electron-holes, which can provide the active substance needed for oxidation reaction. Raja et al.^66^ synthesized rGo–BiVO_4_–ZnO to degrade CIP. 

The formation of ternary heterojunction shortens the transfer distance of electron hole pairs, and the catalytic efficiency will be greatly improved. GF is an ideal photocatalytic material due to its specific surface area, mechanical strength and high active site. Orimolade et al.^67^ prepared n–n heterojunction BiVO_4_/MnO_2_ by electrodeposition on FTO glass, and measured the degradation rate of CIP was up to 76%. Electron microscopy showed that BiVO_4_ particles were embedded in the gaps of the MnO_2_ film^68^, and the Fermi level aligned when the two semiconductors were in contact^69^, which was helpful for the separation of photogenerated carriers. The material allowed that the piperazine ring of CIP can be oxidized and decomposed in a short time, and the ·OH produced by the reaction with water can also degrade CIP indirectly.

**Figure 5:**
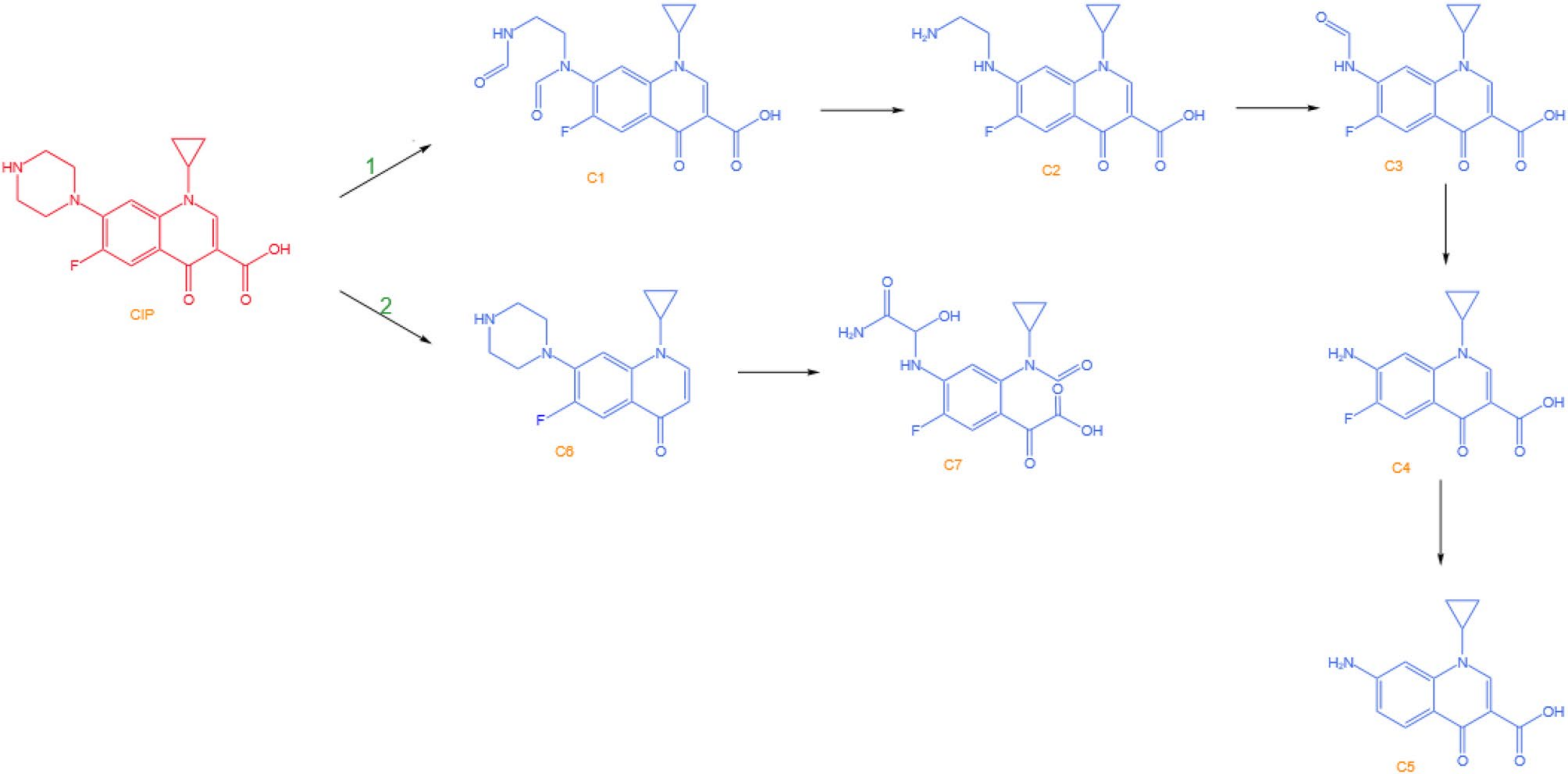
CIP degradation mechanism^25^_._ Copyright 2019 Chemical Engineering Journal.

Analysis of CIP degradation mechanism^25^: the degradation of CIP is mainly due to the cleavage of the piperazine ring and cyclopropyl in the quinolone structure by ·OH to generate CO_2_ and H_2_O, as shown in Fig. 5. Approach 1 is the attack of ·OH on the piperazine ring, which undergoes decarboxylation and hydroxyl substitution to produce C2 and C3. Approach 2 is the cleavage of the quinolone ring by h^+^, which undergoes decarboxylation to convert to C6, followed by the formation of a carboxylic acid group and cleavage of the piperazine ring. Finally, the CO group is eliminated to produce CO_2_, H_2_O and inorganic acid.

#### Ofloxacin (OFL)

OFL is mainly used in gram-positive cocci. When the pH increases to 7, the positive point carried by the OFL piperazine ring combines with the negative charge on the compound, then a redox reaction occurs to cleave the piperazine ring^69^.

Zhao et al.^70^ prepared the Z-scheme BiVO_4_/gC_3_N_4_/NiFe_2_O_4_. In the setting of Z scheme, electrons in gC_3_N_4_ and NiFe_2_O_4_CB are difficult to combine with holes in BiVO_4_VB, which enhances the degradation of OFL by this material. The results show that when the mass fraction of NiFe_2_O_4_ is 5%, the degradation rate of OFL is the highest, up to 93.8%. However, the introduction of metallic elements will increase non-radiative transition centers and intrinsic defects, and reduce light absorption^55^. For example, a relatively high proportion of NiFe_2_O_4_ will promote the reduction of Fe^3+^ to Fe^2+^, leading to the occupation of BiVO_4_ surface active centers, thus hinders photoexcitation and inhibits photocatalytic activity^71^.

Beyond that, the experiment also proves that NiFe_2_O_4_ exhibits better performance in the recovery of catalyst after the reaction due to its magnetic properties^71^. Wen et al.^72^ explored the high efficient catalysis of BiVO_4_/CQDs/β-FeOOH to ofloxacin, and CQDs has good electron storage and transfer ability, which is suitable for electron transfer channel. β-FeOOH provides long-lasting catalytic activity due to its renewable empty oxygen vacancies. XPS was used to measure the complex state before and after the reaction, and by this it was found that electron transfer occurred between divalent iron, ferric iron and oxygen atoms, resulting in larger oxygen vacancies on the surface of the compound, which is conducive to the chemisorption and activation of O_2_ and H_2_O^73^. At the same time, the composites showed the weakest fluorescence intensity when β-FeOOH was added, suggesting that the enhanced separation of electron hole pairs resulted from the formation of heterojunctions between β-FeOOH and BiVO_4_/CQDs specific degradation mechanism of NOR.

**Figure 6:**
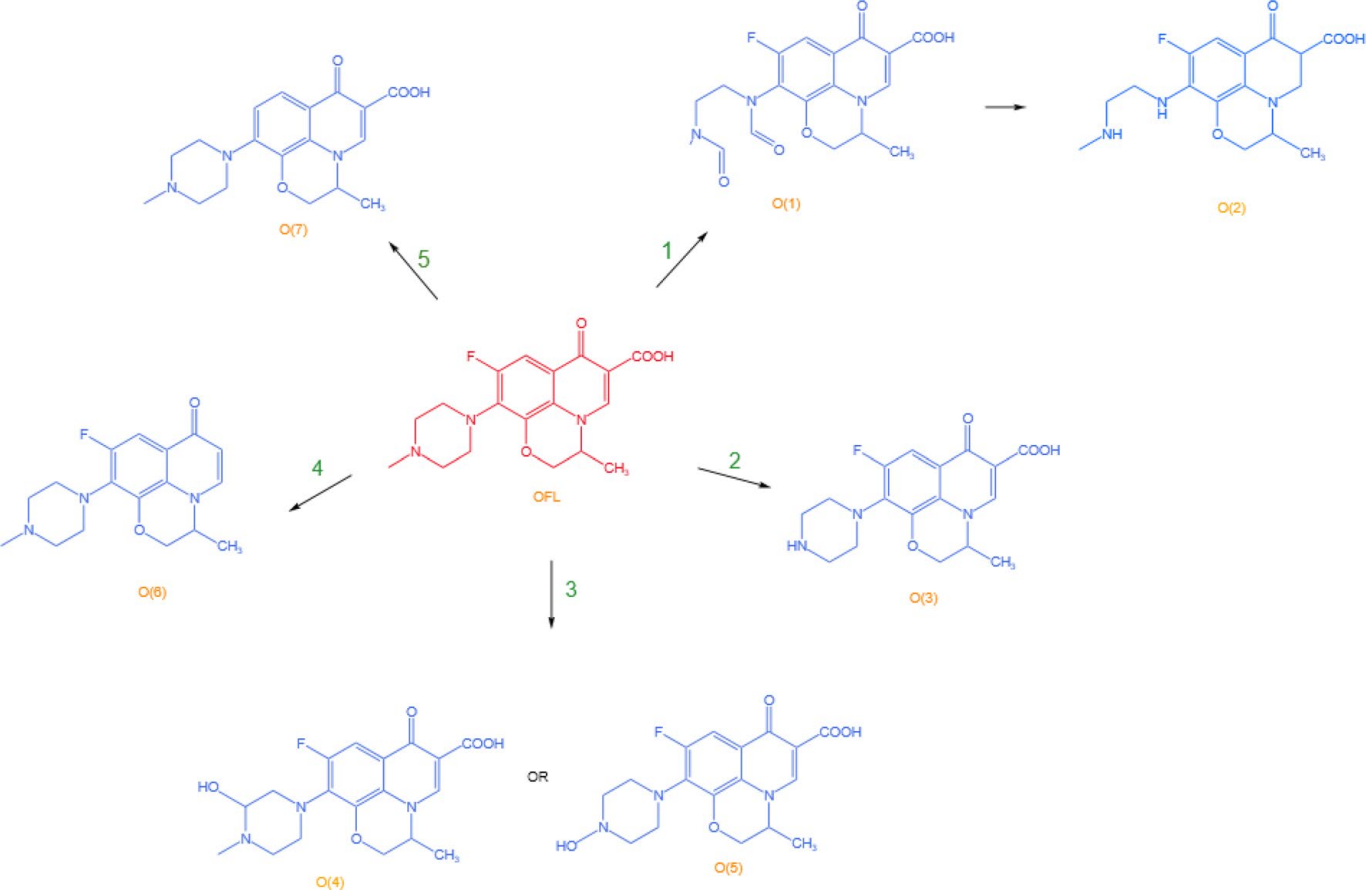
OFL Degradation mechanism.

The degradation mechanism of OFL is shown in Fig. 6, in which involved the main ring-opening reactions^70,74^, piperazinyl demethylation^70,74^, hydroxylation reactions^70,75^, decarboxylation reactions^70^, and defluorination reactions^74^. Approach 1 is that ·O_2_^−^ attacks the piperazine ring to make it cleaved, and the acetyl group is unstable to react further to produce O2. Approach 2 is that ·O_2_^−^ and h^+^ attacked piperazinyl substituent in OFL to achieve demethylation process to produce O3. Approach 3 is hydroxylation reaction, which can occur on the piperazine ring to produce O4 or a combination of demethylation and hydroxylation to generate O5. As for approach 4 the decarboxylation occurred directly to produce O6. Approach 5 is the defluorination reaction to generate O7.

In the past decades, photocatalysis based on BiVO_4_ composites has been widely used for the treatment of antibiotics in water due to its unique narrow band gap as well as the energy-efficient and environmentally friendly nature of the catalysis (see Table 2). However, various problems need to be solved to convert BiVO_4_ composites directly into commercial products.

**Table 2:** Photocatalysts for the degradation of quinolones.

Quinolone	Photocatalyst	Degrading free radicals	Catalytic conditions	Degradation rate %	Ref
LFX	FeVO_4_ /BiVO_4_	·OH/ h^+^ > ⋅O_2_^−^	Sonophotocatalytic; 60 min Catalyst 500 mg/L; LFX 20 mg/L	98.91	^34^
	BiVO_4_ -CeVO_4_	·OH > h^+^ > ·O_2_^-^	Visible light photocatalytic; 300 min Catalyst 500 mg/L; LFX 50 mg/L	95.7	^76^
	Ag_3_ PO_4_ /BiVO_4_	·OH > ·O_2_^-^ > h^+^	Visible light photocatalytic; 300 min Catalyst 1000 mg/L; LFX 10 mg/L	92.4	^77^
NOR	Ag_3_ PO_4_ /BiVO_4_	·OH > ·O_2_^-^ > h^+^	Photoelectrocatalytic; 90 min Applied bias 0.5 V; NOR 5 mg/L	100	^78^
	BiVO_4_/GQDs/PCN	·OH/ h^+^ > ⋅O_2_^−^	Visible light photocatalytic; 120 min Catalyst 1000 mg/L; NOR 20 mg/L	86.3	^63^
	BiVO_4_ /WO_3_	h^+^ > ·O_2_^−^/·OH	Photoelectrocatalytic; 180 min Applied bias 1.0 V; NOR 10 mg/L	67	^79^
	BiVO_4_ /BiOBr	·O_2_^−^/·OH	Visible light photocatalytic; 120 min Catalyst 500 mg/L; NOR 5 mg/L	75	^80^
	M-BiVO_4_/T-BiVO_4_	h^+^ > ·O_2_^−^/·OH	Visible light photocatalytic; 150 min Catalyst 1000 mg/L; NOR 20 mg/L	91	^37^
CIP	rGO-BiVO_4_ -ZnO	h^+^ > ·O_2_^−^/·OH	Visible light photocatalytic; 60 min Catalyst 300 mg/L; CIP 4 × 10^–5^ M	98.4	^81^
	rGO-BiVO_4_	·OH/·O_2_^−^ > h^+^	Visible light photocatalytic; 60 min Catalyst 200 mg/L; CIP 10 mg/L	68.2	^82^
	CuS/BiVO_4_	h^+^ > ·O_2_^−^/·OH	Visible light photocatalytic; 90 min Catalyst 1000 mg/L; CIP 10 mg/L	86.7	^25^
	Fe-BiVO_4_	h^+^ > ·O_2_^-^/·OH	UV–Vis light photocatalytic; 30 min Catalyst 200 mg/L; CIP 10 mg/L	100	^83^
	Ag@PCNS/ BiVO_4_	h + /·O_2_^-^ > ·OH	Visible light photocatalytic; 120 min Catalyst 1000 mg/L; CIP 10 mg/L	92.6	^84^
	FTO/BiVO_4_ /MnO_2_	·OH/ h^+^ > ⋅O_2_^−^	Photoelectrocatalytic; 120 min Applied bias 1.5 V; CIP 10 mg/L	76	^67^
OFL	BiVO_4_ /g-C_3_N_4_ /NiFe_2_O_4_	·OH/ h^+^	Visible light photocatalytic; 20 min Catalyst 1000 mg/L; OFL 10 mg/L	93.8	^70^
	BiVO_4_ /CQDs/β-FeOOH	⋅O_2_^−^	Visible light photocatalytic; 15 min Catalyst 600 mg/L; OFL 10 mg/L	99.21	^72^

(1) The laboratory cannot adequately mimic the real water environment.There are many anions in the sewage, and these ions can reduce the degradation efficiency of antibiotics by redox reactions with active substances (Eqs. (1–4))^70^.

1 $${\text{Cl}}^{ - } +^{\cdot} {\text{OH }} \to {\text{ Cl}}^{\cdot} + {\text{ OH}}^{ - }$$

2 $${\text{NO}}^{{3^{{^{ - } }} }} + {\text{h}}^{ + } \to {\text{ NO}}3^{\cdot}$$

3 $${\text{SO}}_{{4}}^{{{{2}{^{ - } }} }} +^{\cdot} {\text{OH}} \to {\text{ SO}}_{{4}}^{\cdot - } + {\text{ OH}}^{ - }$$

4 $${\text{HCO}}^{{{{3}{^{ - } }} }} + {\text{h}}^{ + } \to {\text{ CO}}_{{3}}^{\cdot - } + {\text{ H}}^{ + }$$

Therefore, there is a need to promote the production of oxidizing substances while reducing their unnecessary consumption. Besides, selective adsorption of different pollutants can be performed by changing the pore size of the composite.

(2) In the process of catalytic degradation of antibiotics, there is a possibility of incomplete degradation. Take NOR as an example, the intermediate (INH) formed by its incomplete degradation has strong toxicity and will form new oxygenated organic compounds when it stays for a long time^55^, which may be more contaminating than the maternal antibiotic. Therefore, the rational design of the BiVO_4_ composites and the improvement of its oxidative properties are necessary^63^.

(3) Since the degradation of quinolones is essentially protonation and deprotonation, the charge on the surface of quinolones depends largely on the pH of the solution^54^. Its influence is mainly reflected in the following two aspects: (1) The choice of pH easily affects the adsorption capacity and chemical structure of the catalyst. Under the environment of strong acid and alkali, the degradation efficiency is extremely low due to the strong electrostatic repulsion generated. (2) When the pH is alkaline, the by-product HCO_3_^2−^ produced during the re-decomposition degrades ·OH and hinders the process of the reaction; when the PH is acidic, HCO_3_^2−^ will interfere with the reactants by escaping from the solution as the form of CO_2_, which is one of the reasons why the photocatalytic reaction is generally carried out under acidic conditions. However, the structure of BiVO_4_ is unstable under acidic conditions, which makes it prone to be slightly soluble, and the Bi dissolved in the aqueous body can become a contaminant that is difficult to remove. Beyond that, different antibiotics have different PH requirements for degradation. Most antibiotics are more easily degraded in acidic environments due to the fact that the main degradation active substance is ·OH. But some organics, such as OFL, whose main substance degraded is·O_2_^−^, are mostly found in alkaline environments^26^. Therefore, there is still a need to optimize the structure and properties of BiVO_4_ composites in the future.

(4) Illumination is the necessary condition for the photocatalytic degradation of BiVO_4_, which means that continuous visible light is required for the degradation process. Nevertheless, in the actual diurnal process, visible light is not continuously available, making the application limited^76^. Hence, low-cost light storage devices need to be developed in the future to ensure the reaction.

## Summary and outlook

In summary, BiVO_4_ photocatalytic composites have good photocatalytic properties and can be widely used to treat environmental pollutants. The BiVO_4_ composites built by noble metal deposition, elemental doping, and heterostructure have showed good performance in quinolone degradation.

Meanwhile, the degradation mode can be optimized by adding ultrasound, voltage and Fenton techniques to the conventional photocatalysis. However, there are still many issues to be debated and solved in the practical application of BiVO_4_ composites. Current researches on composites have focused more on the physicochemical structure of the material itself, and then neglect the nature of the degraded products and the relationship with photocatalytic composites. In addition, the degradation process is faced with the contamination caused by the complex properties of antibiotics and BiVO_4_ itself. The research of BiVO_4_ photocatalytic composites is still in the study stage, while the real use of degradable substances such as antibiotics is still quite lacking, which needs to be further related to the actual practice in order to improve the production method, enhance the manufacturing efficiency and reduce the production cost. BiVO_4_ photocatalytic composites have a broad research prospect and will be widely used in various fields in the future.

## Data Availability

The authors confirm that the data supporting the findings of this study are available within the article.
